# Allosteric Interactions between the Myristate- and ATP-Site of the Abl Kinase

**DOI:** 10.1371/journal.pone.0015929

**Published:** 2011-01-10

**Authors:** Roxana E. Iacob, Jianming Zhang, Nathanael S. Gray, John R. Engen

**Affiliations:** 1 Department of Chemistry & Chemical Biology, and The Barnett Institute for Chemical and Biological Analysis, Northeastern University, Boston, Massachusetts, United States of America; 2 Biological Chemistry & Molecular Pharmacology, Harvard Medical School and Department of Cancer Biology, Dana-Farber Cancer Institute, Boston, Massachusetts, United States of America; University Paris Diderot-Paris 7, France

## Abstract

Abl kinase inhibitors targeting the ATP binding pocket are currently employed as potent anti-leukemogenic agents but drug resistance has become a significant clinical limitation. Recently, a compound that binds to the myristate pocket of Abl (GNF-5) was shown to act cooperatively with nilotinib, an ATP-competitive inhibitor to target the recalcitrant “T315I” gatekeeper mutant of Bcr-Abl. To uncover an explanation for how drug binding at a distance from the kinase active site could lead to inhibition and how inhibitors could combine their effects, hydrogen exchange mass spectrometry (HX MS) was employed to monitor conformational effects in the presence of both dasatinib, a clinically approved ATP-site inhibitor, and GNF-5. While dasatinib binding to wild type Abl clearly influenced Abl conformation, no binding was detected between dasatinib and T315I. GNF-5, however, elicited the same conformational changes in both wild type and T315I, including changes to dynamics within the ATP site located approximately 25 Å from the site of GNF-5 interaction. Simultaneous binding of dasatinib and GNF-5 to T315I caused conformational and/or dynamics changes in Abl such that effects of dasatinib on T315I were the same as when it bound to wild type Abl. These results provide strong biophysical evidence that allosteric interactions play a role in Abl kinase downregulation and that targeting sites outside the ATP binding site can provide an important pharmacological tool to overcome mutations that cause resistance to ATP-competitive inhibitors.

## Introduction

Protein kinases are now avidly pursued as therapeutic targets for a host of human ailments, especially cancers [1]–[2]. The vast majority of reported inhibitors target the ATP binding site but because the ATP binding pocket is highly conserved among the human protein kinase, there can be cross-reactivity with a number of other kinases. This cross-reactivity is, in many cases, therapeutically undesirable. The search for more potent and target-specific ATP site inhibitors has been met with limited success making alternative kinase inhibition approaches involving therapeutics that target sites other than the ATP binding pocket very attractive. As many protein kinases have multiple regulatory sites that are often kinase specific, these sites provide the opportunity to develop non-ATP competitive protein kinase inhibitors with potentially higher selectivity.

Abl kinase is an important inhibitor target due to the role of the Bcr-Abl fusion protein in the development of Chronic Myleogenous Leukemia (CML). Imatinib (STI-571, Gleevec) [3], nilotinib (AMN 107) [4] and dasatinib (BMS-354825) [5] are among the ATP-competitive inhibitors of Bcr-Abl catalytic activity that have demonstrated remarkable efficacy in chronic-phase CML (reviewed in [6]–[9]). For example, imatinib results in a greater than 80% response rate when patients are treated in the chronic phase of CML. However, approximately 60% of patients in the blast-crisis phase will develop resistance to imatinib [10]–[12]. Drug resistance can occur upon the emergence of cells expressing point mutations in Bcr-Abl [9]. Of the more than 50 clinically detected point mutations in Bcr-Abl, the majority occur in the ATP-binding pocket and appear to result in a steric impediment to drug binding [11], [13]–[15]. Other mutations remote from the ATP-binding site are thought to confer resistance by destabilizing the “DFG-out” conformation required for imatinib binding [5] or thorough other allosteric mechanisms. Later generation inhibitors such as nilotinib, dasatinib and bosutinib [16] overcome some of the resistance created by the majority of the mutations. Both dasatinib and nilotinib exhibit higher binding affinity for the ATP-site and can overcome all but the T315I gatekeeper mutation [4], [17]. In addition, other new ATP-competitive inhibitors capable of inhibiting T315I Bcr-Abl have been reported in conjunction with co-crystal structures: PPY-A [15], SGX393 [18], and PHA-739358 [14], AP24163 [19], DSA series compounds [20], HG-7-85-01 [21] and AP24534 [22]; see also [23].

We previously reported on the discovery of GNF-2, a small molecule inhibitor of Bcr-Abl dependent cell proliferation [24]. Based upon mutational analysis, GNF-2 was found to bind not to the ATP pocket, but instead to the myristate binding pocket located at the C-terminus of the Abl kinase domain. Studies with drug resistant mutants showed that GNF-2 maintains potency against a subset of the clinically relevant imatinib-resistant Bcr-Abl mutants (e.g., E255V, Y253H), but was surprisingly much weaker against the T315I gatekeeper mutant [24]. Further evidence showed that GNF-2 compounds do indeed bind to the myristate pocket [25] and effectively inhibit kinase activity on their own.

In the current work, we set out to understand mechanistically how GNF compounds inhibit kinase activity. In addition to potentially changing the conformation of the αI helix, GNF-2 binding could allosterically influence the catalytic site resulting in kinase inhibition through an as of yet unknown mechanism. This hypothesis has validity because there appears to be more to the effect of the GNF inhibitor than enhancement of SH2 docking to the kinase domain. GNF-2 and GNF-5 are not potent cellular inhibitors of the T315I mutant but combinations of nilotinib [25] or dasatinib (below) with GNF-5 are very effective inhibitors of T315I both *in vitro* and *in vivo*. The ability of the T315I mutation to activate Abl [26] and the inability of T315I to be potently inhibited by GNF-2/5 suggests that this mutation may cause a more widespread conformational rearrangement of the kinase domain that partially uncouples it from allosteric control by the myristic acid pocket in the large lobe. In addition, because other sites of both activating mutations and inhibitor binding can occur well outside of the Abl active site, there seems to be evidence supporting the existence of long-range conformational coupling within the Abl kinase core. Therefore, allosteric inhibitors that target the Abl kinase core may function by altering the conformational dynamics of the active site and its ability to bind to ATP competitive inhibitors.

Hydrogen exchange (HX) mass spectrometry (MS) was used to study the conformational effects that result from binding of dasatinib and GNF-5, both individually and in combination, to wild-type Abl core (a construct containing the NCap, SH3, SH2 and the kinase domain) and a T315I mutant version of Abl core. All experiments were performed with the Abl core expressed in *E. coli* [27]; therefore, this protein was not myristoylated on the N-terminus and it is in an active conformation, as was shown previously [27]. While many drug-interaction studies have been performed with the kinase domain alone, the present results are some of a few conformational studies that make use of the entire Abl kinase core.

The results show that dasatinib binding to Abl induced dynamic changes near the ATP binding site only. There were no significant changes in hydrogen exchange for Abl T315I in the presence of dasatinib, illustrating a lack of interaction between dasatinib and this mutant. As shown previously, GNF-5 binding to the myristic acid site caused a reduction of hydrogen exchange in both the myristate and the ATP site [25]. GNF-5 still elicited the same alterations to hydrogen exchange in T315I as it did in wt Abl. Finally, in a combination experiment, hydrogen exchange in the T315I protein in the presence of both dasatinib and GNF-5 demonstrated changes in exchange near the ATP site that were nearly the same as those seen when dasatinib bound to wild-type Abl. Taken together, the results provide evidence for allosteric communication between the two sites and indicate that the factors leading to dasatinib resistance are somehow overcome by simultaneous binding of GNF-5 some 25 Å away. Targeting the Abl myristate binding site can provide an important pharmacological tool to suppress the rise of drug resistant mutations, and overcome existent mutations that are resistant to ATP-competitive inhibitors.

## Materials and Methods

### Materials

96% formic acid, deuterium oxide (99.9%), sodium chloride and Tris-hydrochloride were purchased from Sigma-Aldrich (St. Louis, MO). Acetonitrile and water (W5-4 HPLC grade) were purchased from Fisher Scientific. Potassium phosphate was purchased from EMD Biosciences. All chemicals were used without further purification unless otherwise specified. GNF-5 [25] was synthesized according to published procedures and dasatinib was purchased from LC Laboratories (Woburn, MA).

### Preparation of recombinant proteins

The bacterial expression of human c-Abl kinase (residues 46–515, Abl 1a numbering [28]) was performed as previously described [29]. A pET-28a encoded Abl was co-expressed with YopH phosphatase in Escherichia coli BL21DE3 cells. Cultures were grown to an OD_600_ of 1.2 at 37°C, cooled for 1 h with shaking at 18°C prior to induction for 16 h at 18°C with 100 µM IPTG. Cells were harvested by 10 min centrifugation at 7000 g at 4°C and resuspended in 50 mM Tris (pH 8.0), 500 mM NaCl, 5% glycerol, 25 mM imidazole (buffer A). Insoluble protein and cell debris of cell lysate were sedimented by centrifugation at 40,000 g for 40 minutes at 4°C. The supernatant was loaded onto a Ni affinity column (HisTrap FF, GE Lifescience), equilibrated with buffer A. The loaded column was washed with five column volumes of buffer A, and protein was eluted with a linear gradient of 0–50% of buffer B (Buffer A plus 0.5 M imidazole). The peak fractions were analyzed by SDS-PAGE. Fractions containing the kinase were pooled and dialyzed against 20 volumes of buffer QA (20 mM Tris-pH 8.0, 100 mM NaCl, 5% glycerol, 1 mM DTT) using a 13-kDa molecular weight cutoff membrane. Tobacco etch virus protease TEV (expressed in bacteria using a plasmid kindly provided by M. J. Eck lab at DFCI) was added to the pooled protein to cleave the His tag (the ratio of TEV to total protein was 1 to 60). Dialysis and protease digestion were carried out overnight at 4°C. Subsequent anion exchange chromatography (HiTrap Q FF, GE Lifescience) at room temperature was used to remove protease and phosphatase contaminants. Proteins were eluted with a linear gradient of 0–35% buffer QB (buffer QA plus 1 M NaCl), and peak fractions were analyzed by SDS-PAGE.

Purity and mass of all proteins were verified by electrospray mass spectrometry (see Supplemental Figure S1). The proteins were not myristoylated or Ser69 phosphorylated. No inhibitors or ATP/Mg^2+^ were used during purification of Abl proteins or data acquisition. See Ref. [27] for amino acid sequence information.

### Kinetic characterization of Abl inhibition

The ATP/NADH-coupled assay system in a 96-well format was used to determine the initial velocity of Abl tyrosine kinase catalyzed peptide phosphorylation. The reaction mixture contained 20 mM Tris-HCl, (pH 8.0), 50 mM NaCl, 10 mM MgCl_2_, 2 mM PEP [2-(Phosphonooxy)- 2-propenoic acid, Sigma-Aldrich, cat. P-7002) and 20 µM Abl peptide substrate (EAIYAAPFAKKK, New England Biolabs, Cat No. P6051L), fixed or varied (to determine inhibitor kinetic parameters) concentration of inhibitor applied, 1/50 of the final reaction mixture volume of PK/LDH enzyme (pyruvate kinase/lactic dehydrogenase enzymes from rabbit muscle, Sigma-Aldrich, cat. P-0294), 160 µM NADH, 0.16 µM Abl, and ATP added last to start the reaction. Absorbance data were collected every 20 s at 340 nm using a SpectraMax M5 Microplate Reader. The two-substrate kinase reaction was simplified to two one-substrate reactions to determine ATP kinetic parameters and inhibitor parameters separately. When determining ATP parameters, the inhibitor concentration was kept the constant. When determining inhibition parameters, the ATP concentration was fixed at 20 µM. Steady-state initial velocity data were drawn from the slopes of the A340 curves and fit to the Michaelis-Menten equation to determine V_max_ and K_m_ values. Data were fitted globally using GraphPad Prism (GraphPad Software) and Excel XLfit 4.0 to fit velocity equations for competitive and mixed inhibition. At a concentration of 3 uM, GNF-5 does not achieve 100% inhibition of Abl. A concentration range of 0–3 µM was the range where positive additive effects of GNF-5 and dasatinib were apparent. Full inhibition of WT Abl could be achieved at concentrations of in excess of 3 uM.

### Cell proliferation assay and measurement of resistant colony formation

4×10^3^ wildtype or Bcr-Abl transformed Ba/F3 cells per well were plated in duplicate in 384-well plates, in RPMI 1640 media supplemented with 10% FBS, (addition of 10 ng/ml of IL-3 for wide type Ba/F3 cells). Test compounds in DMSO stocks were serially diluted in DMSO and added using a 384 pin tool. After 48 h of growth, Bright-Glo reagent (Promega, WI) was added to each well to determine cell viability as a percentage of growth in the absence of the compound. Luminescence was read as counts/sec. XL-fit (IDBS) was used for IC_50_ analysis. For the resistant colony formation experiment, cultures containing 1×10^4^ Ba/F3 cells in 96-well plates were incubated with various concentrations of GNF-5, dasatinib or both. The cells were continuously exposed to compound in RPMI 1640 with 10% FBS medium and medium was changed every three days with fresh inhibitor added each time. The number of resistant colonies was counted at day 12.

### Hydrogen exchange experiments

Hydrogen exchange experiments were performed essentially as described in Iacob et al [27]. Prior to the addition of deuterium, Abl was allowed to equilibrate with each drug. GNF-5 and dasatinib were in 100% DMSO at a concentration of 10 mM. 38 pmol of each protein was incubated with 85 µM dasatinib and GNF-5 for a protein:inhibitor ratio of 1∶7. For a K_d_ of 0.1 µM, 98% of the proteins were bound to the inhibitor. All mixtures were incubated for 30 min at room temperature before deuterium labeling. As a control, proteins were incubated in 20 mM Tris, 100 mM NaCl (pH 8.3) buffer and treated exactly as the inhibitor- bound proteins.

Deuterium exchange was initiated by dilution of the free or bound protein with 15-fold 20 mM Tris, 100 mM NaCl (pD 8.3), D_2_O buffer at room temperature. At each deuterium exchange time point (from 10 s to 4 hours) an aliquot from the exchange reaction was removed and labeling was quenched by adjusting the pH to 2.6 with an equal volume of quench buffer (50 mM potassium phosphate, pH 2.6, H_2_O). Quenched samples were immediately frozen on dry ice and stored at −80°C until analysis. The same procedure was used for the labeling of all proteins used in this study.

Each frozen sample was thawed rapidly to 0°C and injected into a custom Waters nanoACQUITY UPLC system and analyzed as described previously [30]. The protein sample was digested using a Poroszyme immobilized pepsin cartridge (Applied Biosystems) which was accommodated within the UPLC system. The cooling chamber of the UPLC system, which housed all the chromatographic elements was held at 1°C for the entire time of the measurements. The injected peptides were trapped and desalted for 3 min at 100 µL/min and then separated in 6 min by an 8%–40% acetonitrile:water gradient at 40 µL/min. The separation column was a 1.0×100.0 mm ACQUITY UPLC C18 BEH (Waters) containing 1.7 µm particles and the back pressure averaged 8800 psi at 1°C. The average amount of back-exchange using this experimental setup was 18%–25%, based on analysis of highly deuterated peptide standards. Deuterium levels were not corrected for back-exchange and are therefore reported as relative [31]; however, all comparison experiments were done under identical experimental conditions thus negating the need for back-exchange correction [31]. The UPLC step was performed with protonated solvents, thereby allowing deuterium to be replaced with hydrogen from side chains and the amino/carboxy terminus that exchange much faster than amide linkages [32]. All experiments were performed in triplicate. The error of determining the deuterium levels was +/−0.20 Da in this experimental setup.

Mass spectra were obtained with a Waters QTOF Premier equipped with standard ESI source (Waters Corp., Milford, MA, USA). The instrument configuration was the following: capillary was 3.5 kV, trap collision energy at 6 V, sampling cone at 37 V, source temperature of 100°C and desolvation temperature of 250°C. Mass spectra were acquired over an *m/z* range of 100 to 2000. Mass accuracy was ensured by calibration with 100 fmol/µL GFP, and was less than 10 ppm throughout all experiments. The mass spectra were processed with the software HX-Express [33] by centroiding an isotopic distribution corresponding to the +2, +3, or +4 charge state of each peptide. In HX-Express, deuteration levels were calculated by subtracting the centroid of the isotopic distribution for peptide ions of undeuterated protein from the centroid of the isotopic distribution for peptide ions from the deuterium labeled sample. The resulting relative deuterium levels were plotted versus the exchange-in time. Identification of the peptic fragments was accomplished through a combination of exact mass analysis and MS^E^ [34] using custom Identity Software from the Waters Corporation. MS^E^ was performed by a series of low-high collision energies ramping from 5–25 V, therefore ensuring proper fragmentation of all the peptic peptides eluting from the LC system.

## Results and Discussion

Resistance to ATP-competitive kinase inhibitors limits the duration of response that can be achieved with this class of therapeutics. The most common resistance mechanism involves the acquisition of mutations in the ATP-binding site that prevent drug binding or that increase the affinity for ATP. The most frequent site of mutation occurs to the so-called gatekeeper residue which is located towards the rear of the ATP-binding cleft. Many clinically relevant kinases such as Bcr-Abl, c-Kit, EGFR and PDGFR possess a threonine residue at the gatekeeper position that is involved in critical interactions with ATP competitive drugs. Mutation of this residue to a large hydrophobic amino acid such as isoleucine or methionine disrupts inhibitor binding without disrupting the kinase activity of the enzyme. The T315I gatekeeper mutation is resistant to the three Bcr-Abl inhibitors that are currently approved for the treatment of Chronic Myelogenous Leukemia: imatinib, nilotinib and dasatinib. There are two approaches for developing new drugs that can overcome ATP-site mutations such as the gatekeeper mutation. The first is to make new ATP-competitive inhibitors that exploit binding modes that can circumvent the existing mutations. This approach has been successfully exploited and several ATP-competitive T315I Bcr-Abl inhibitors are currently in preclinical development [23]. The weakness of this approach is that there is always likely to be a particular mutation that will be disruptive to a given inhibitor. The second approach is to find allosteric inhibitors that are able to inhibit kinase activity by binding to sites remote from the ATP-site. Allosteric inhibitors have been discovered for a number of kinases [2], [35]–[36]. For example, Bcr-Abl can be effectively inhibited by compounds that bind to the myristate-binding site such as GNF-2 and GNF-5. Allosteric inhibitors have the potential advantage that they can potentially be used in combination with ATP-competitive inhibitors to achieve greater inhibition of the target and make it more difficult to develop a single mutation that reverses drug efficacy. Indeed, GNF-5 was recently demonstrated to act additively with nilotinib to suppress the emergence of resistant Bcr-Abl alleles and to inhibit T315I Bcr-Abl.

**Figure 1:**
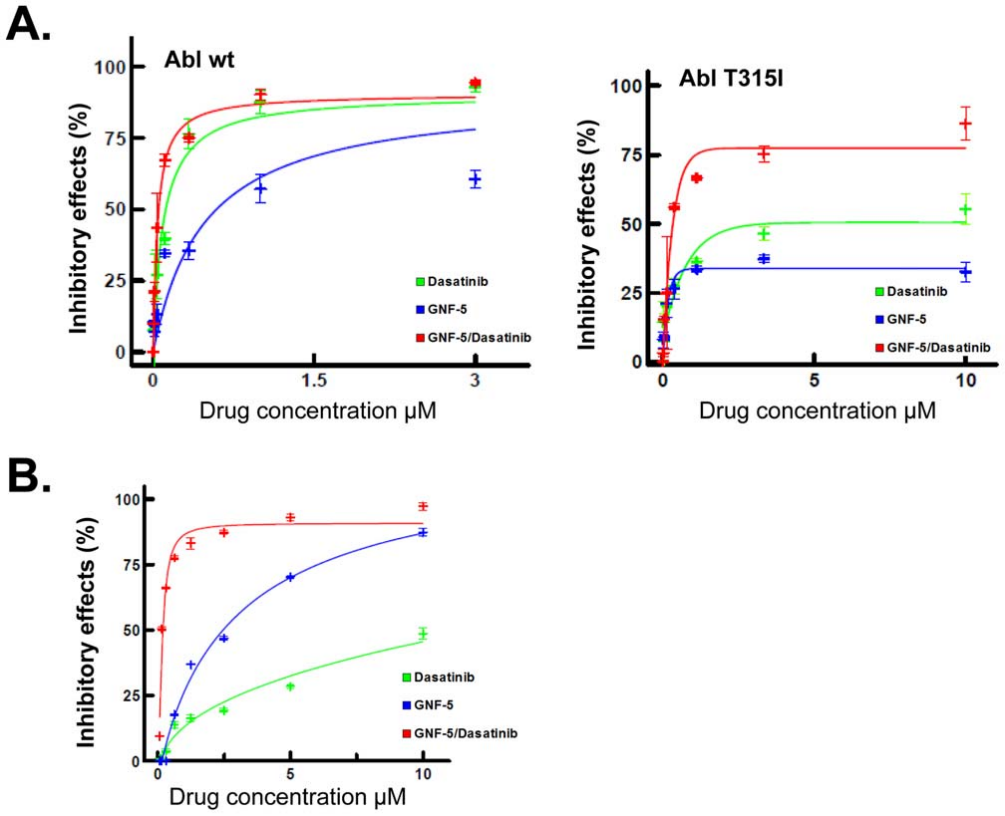
Inhibition of Abl proteins with compounds alone or in combination. **A**. Enzymatic inhibition (*in vitro*) of recombinant wild-type and T315I Abl by dasatinib, GNF-5 and combination treatments. Percent inhibition of wt Abl or T315I Abl by dasatinib and GNF-5 or the combination. The combination curve (red) contains twice the total drug concentration of the single agent curves due to both drugs being present. **B**. Synergistic inhibition of Bcr-Abl T315I transformed Ba/F3 cells. Dose and effect curve of GNF-5, dasatinib and the combination of GNF-5 and dasatinib (1∶1 ratio) on Bcr-Abl T315I transformed cells. The combination curve (red) contains twice the total drug concentration of the single agent curves due to both drugs being present. **C**. CI values for fractional growth inhibitions of 0.50, 0.75, and 0.90 in Bcr-Abl T315I cells. Antagonism CI >1.00; additivity CI = 1.00; synergy CI <1.00.

Here we sought to determine whether cooperative inhibition of wild-type and T315I Bcr-abl could be achieved between the clinically approved inhibitor dasatinib and GNF-5. Dasatinib is an ATP-competitive inhibitor but it binds to Bcr-Abl in the active ‘type I’ conformation in contrast to nilotinib which binds to the inactive conformation. We first evaluated the ability of dasatinib and GNF-5 (or combinations) to inhibit recombinant Abl using a pyruvate kinase-lactate dehydrogenase detection system, that couples the production of ADP to oxidation of NADH which can be monitored spectrophotometrically [37]. Wild-type, T315I, and E505K Abl kinases were expressed in bacteria. Inhibition of wild-type Abl was observed for both inhibitors (Fig. 1A) with GNF-5 exhibiting an IC_50_ = 0.22 µM, dasatinib IC_50_ = 0.12 µM using an ATP concentration of 20 µM, which is close to the apparent K_m_ under our assay conditions. The myristate site mutant E505K was inhibited by dasatinib with an IC_50_ = 0.02 µM, but not by GNF-5 (IC_50_ >10 µM). The T315I mutant was partially inhibited by dasatinib or GNF-5 in this assay. We next examined whether combinations of GNF-5 and dasatinib resulted in additive inhibition of wild-type, T315I, or E505K recombinant Abl proteins. Strong positive cooperativity was observed for combinations of GNF-5 and dasatinib on T315I Bcr-Abl with a calculated combination index of 0.34. As expected no additivity was observed with the E505K myristate site mutant.

We next evaluated the ability of the compounds to inhibit the proliferation of wild-type and T315I transformed Ba/F3 cells (Fig. 1B). The proliferation assays demonstrated that greater than 50% inhibition of T315I Bcr-Abl dependent cell growth could be achieved at a range of GNF-5 and dasatinib concentrations. For example, at a fixed GNF-5 concentration of 10 µM, dasatinib inhibits T315I Bcr-Abl-dependent proliferation with an IC_50_ = 20 nM (Supplemental Fig. S2A). The calculated combination index (CI) for GNF-5 and dasatinib in this assay was 0.65, indicating a synergistic interaction. We also tested the effect of the drug combinations on Bcr-Abl and STAT5 phosphorylation by Western blotting. While 10 µM dasatinib was not able to inhibit Bcr-Abl autophosphorylation and STAT5 phosphorylation in T315I Bcr-Abl expressing Ba/F3 cells, the combined treatment with 10 µM dasatinib plus 0.5, 5, or 10 µM GNF-5 significantly blocked T315I Bcr-Abl signaling (Supplemental Fig. S2B).

Moderate synergism was observed for combinations of GNF-5 and dasatinib on the wild-type Abl *in vivo*, with calculated combination indices of 0.69 (data not shown). Much stronger synergism was observed with the T315I form (CI = 0.32), Fig. 1C. As expected no synergy was observed with the E505K myristate site mutant (Supplemental Figure S2), confirming that GNF-5 binding to the myristic acid pocket was responsible for the inhibition [25].

### HX MS shows changes upon inhibitor binding

Confident that biologically there was strong evidence for cooperativity between the two binding sites, we utilized hydrogen exchange mass spectrometry to try to understand how this synergy worked mechanistically. The conformational dynamics of each protein were measured in the presence of various drug combinations. We previously used this methodology to investigate conformational changes in Abl upon removal of the myristic acid group and upon mutation in a down-regulated form of Abl [27].

Both wild-type Abl and the T315I mutant were overexpressed in *E. coli* and purified to homogeneity. The purity and mass of the proteins were verified by electrospray mass spectrometry (Supplemental Fig. S1). Note that these proteins are prepared from bacteria and are not myristoylated on the N-terminus, making them enzymatically active (see also ref. [27]). Prior to analysis of the HX of each protein in the presence of inhibitors, a control experiment was performed in which we compared exchange into wild-type versus the T315I mutant. This was done to provide the baseline exchange for both forms so there would be a reference for comparison when each form was bound to the inhibitors. Wild-type and T315I were independently exposed to deuterium for various periods of time ranging from 10 seconds to 4 hours. The exchange was quenched, each deuterated protein was digested into peptic fragments, and the location and quantity of deuterium was determined by mass spectrometry [27], [31]. Although a large number of peptic peptides (∼50) were followed, only a few indicated changes in exchange (Supplemental Fig. S3) during the time frame of the experiment (4 hours). Data is not shown for regions in which no changes in HX were detected. Interestingly, we observed few changes in T315I conformational dynamics relative to the wild-type form. All changes were consistent with the incorporation of more deuterium in the T315I form compared with wild-type Abl and were primarily restricted to the small lobe of the kinase domain (see Fig. S3).

**Figure 2:**
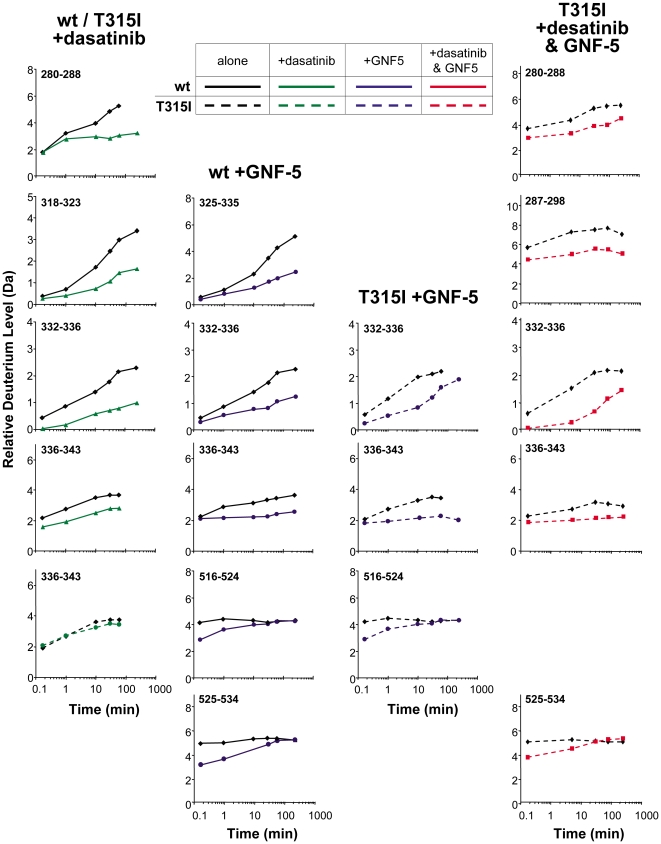
Deuterium incorporation curves for wild-type and T315I Abl in the presence of dasatinib, GNF-5 or both dasatinib and GNF-5. Both dasatinib and GNF-5 were present at a molar ratio of 1∶7, protein:inhibitor. Only data for the peptides that showed changes in deuterium uptake in the presence of inhibitors are shown; all other regions indicated no changes in hydrogen exchange in the presence of the inhibitors in the time frame the experiment was performed (4 hours). Data for free or bound forms were acquired under identical conditions, in duplicate. The error of each data point is ±0.20 Da. Note that we have used Abl 1b numbering [45] throughout this work; for example, Abl position T315 is actually T334 in Abl 1b, but we are using the numbering designation of T315I according to the established clinical conventions [10].

**Figure 3:**
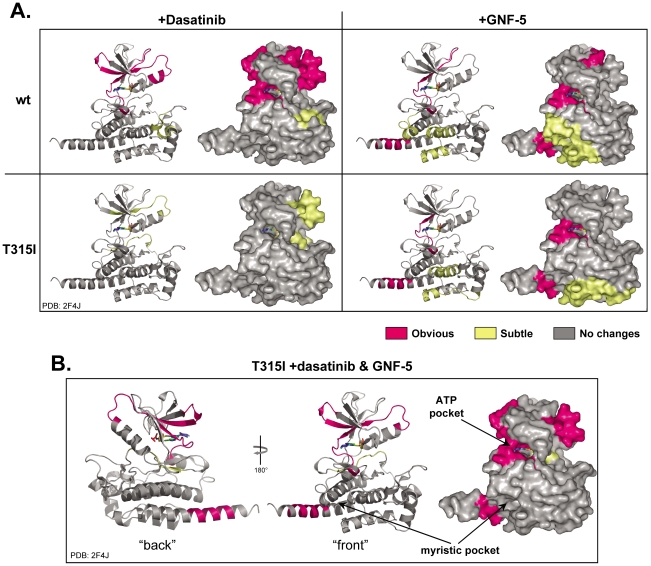
Summary of the hydrogen exchange results for all binding experiments in this study. In each panel, the ribbon diagram of Abl (PDB 2F4J, [46]) is shown in the left and the space filling model is shown on the right. The differences in deuterium levels are colored on each peptide where changes were observed, according to the color code shown. The location of each specific peptide is labeled in Supplemental Fig. S5. Obvious changes (colored hot pink) were defined as a difference between deuterium exchange-in curves of 1.0 Da or more, subtle changes (colored light yellow) were defined as a difference of 0.4–1.0 Da and no changes were defined as differences of 0.0–0.4 Da. The ATP binding site is shown by rendering the drug VX-6, already present in the 2F4J crystal structure. A close-up of the myristic acid binding pocket is shown in Supplemental Fig. S6. Abl structure 2F4J was chosen to interpret the hydrogen exchange data as it has an extended αI helix thought to be present in the active form of Abl. Although the NCap SH3 domain, SH2 domain and the SH2-kinase linker are present in the constructs studied, no changes in hydrogen were detected in those regions in the presence of these inhibitors.

With baseline exchange for both wild type and the T315I proteins, inhibitor binding was probed by HX MS. Each inhibitor was first incubated individually with wild-type Abl or T315I and regions with differences in HX were observed. Again, most peptides indicated no difference in deuterium uptake (data not shown), including all regions of both SH3 and SH2. However a few areas showed differences in deuterium uptake in the presence of dasatinib or GNF-5. The deuterium incorporation data for regions where changes were observed are summarized in Figure 2 (representative examples of the raw mass spectra are shown in Fig. S4). The changes observed upon inhibitor binding cluster into several distinct areas: residues 280–298, 318–343 and 516–524. The three dimensional locations of the changes with dasatinib or GNF-5 and the combination of both are summarized in Fig. 3A and B and a key locating each peptide is provided in Supplemental Fig. S5 (see Ref. [27] for amino acid sequence information). Interpretation of these results is provided in the follow sections.

### Dasatinib causes changes in the small lobe but has no significant effect on T315I

Wild type Abl was incubated with dasatinib in a 1∶7 molar ratio (protein:inhibitor), so that >98% of the Abl molecules were bound, based on a K_d_ of 0.1 µM. The control experiment in which no dasatinib was present was performed at the same time, under identical conditions so the exchange differences could be compared without the need for back-exchange correction (explained in detail in [31]). Changes in deuterium uptake in wild-type Abl in the presence of dasatinib were observed in several peptic peptides, mostly near the ATP binding pocket. For example, the deuterium incorporation graph for the peptide 280–288 indicates that this peptide was less deuterated when dasatinib was bound implying protection from solvent or increased hydrogen bonding in this region. Other regions with changes in the presence of dasatinib included 318–323, 332–336 and 336–343. The cumulative error of measuring deuterium uptake in these assays is approximately ±0.20 Da. Any differences larger than that were considered significant for the purposes of comparing the two datasets. The changes were grouped according to obvious changes (>1.0 Da separating the deuterium incorporation curves, after two replicates were averaged) and subtle changes (0.4–1.0 Da difference).

The location of the changes in wild type Abl when bound to dasatinib are shown in Fig. 3A, top left. The bulk of the changes are surrounding the ATP binding pocket, and are consistent with stabilization of this region upon dasatinib binding. Dasatinib lies in the ATP site with the aminothiazole group occupying the site bound by the adenine group of ATP [38]–[39]. Previous studies identified three notable hydrogen bonds between dasatinib and Abl. One with Met337 (Met318) residue, one with Thr334 (T315) and one with the carbonyl oxygen of Glu335 (Glu316) [38]. Other contacts are mainly van der Waals interactions between the phenyl ring of dasatinib and Leu267 (Leu248), Gly340 (Gly321), Met309 (Met290), Val318 (Val299), Ile332 (Ile313) [38]. The HX MS results are consistent with protection/stabilization of these amino acids, as all changes we detected were protection from deuteration in peptic peptides including these amino acids.

It was shown previously that the P-loop is very flexible even when dasatinib is bound indicating that for the most part the P loop does not form a critical interaction with dasatinib [38], [40]. Based on molecular dynamics studies it has been hypothesized that dasatinib can bind to both the active DFG- in and inactive DFG- out conformations of Abl [38], [41]. The HX MS data on the peptide spanning the P-loop (residues 257–274) illustrated that when dasatinib is bound to Abl, the amount of deuterium uptake in this peptide is the same as in unbound protein, indicating that there are no critical interactions between P-loop and dasatinib (data not shown). This feature may be advantageous because several imatinib resistant Abl mutations appear in the P-loop.

In contrast to what was observed for dasatinib binding to wild type Abl, when dasatinib was incubated at the same molar ratio with the T315I mutant, no major changes in HX were detected. For example, compared with wild-type Abl, residues 336–343 displayed no changes in deuterium incorporation when dasatinib was incubated with T315I (Fig. 2). Subtle changes in the deuterium uptake were detected in residues 287–298 and in two overlapping peptides spanning the DFG motif (396–403 and 402–406), but these changes were relatively minor in comparison with the changes observed in wild-type Abl and dasatinib (see Fig. 3A, Fig. S5). Previously it was indicated that dasatinib is involved in a hydrogen bond with the side chain of T315, and the side chain methyl group of the threonine is involved in van der Waals contacts with the 2-chloro-6-methyl phenyl ring [38]. Upon mutation, these contacts are lost and the bulkier isoleucine side chain increases steric hindrance to dasatinib binding [15]. The HX MS data nicely show (Fig. 3) that the loss of binding can be clearly observed and is consistent with the known inability of dasatinib to inhibit the T315I mutant form of Abl.

### GNF-5 binding to wild-type or T315I Abl induces a distant conformational change

We previously showed that GNF-5, a compound representing a novel class of allosteric Bcr-Abl inhibitors, specifically binds to the myristic acid binding pocket [25]. Upon binding, hydrogen exchange is not only reduced in the myristic acid pocket, but also at a distance in the ATP site. This allosteric effect is specific to GNF-5 binding. Mutation of a residue (E505K, see Supplemental Fig. S6) known to be specific and required for GNF-5 interaction, and the reduction in kinase activity (Fig. S2), abolishes the reduction of HX in the ATP site. We now show that GNF-5 can also elicit the same effects in the T315I mutant.

Both wild-type Abl and the T315I mutant form were incubated with GNF-5 and independently exposed to D_2_O for various periods of time as described above. Changes in deuterium incorporation in the presence of GNF-5 were observed in several peptides (Fig. 2) and the results were highly similar for both wild-type or T315I. The peptides 516–524 and 525–534 correspond to the C-terminal αI-helix in the kinase domain. Both were affected by GNF-5 binding (Figs. 2, 3, S4C) and the changes were the same for both wild-type and T315I. After 10 seconds in deuterium, exchange was reduced by approximately 1 deuterium atom, implying that the effect was a result of steric occlusion rather than long-term changes in protein stability or dynamics [42]–[43]. Furthermore, other peptides located in close proximity to the myristic pocket showed slightly decreased deuterium uptake when the GNF-5 was present (Fig. 3A). These results strongly support previous data which indicate that the location of the GNF-5 is indeed in the myristic pocket [25] and show that GNF-5 binding occurs regardless of the T315I mutation.

Hydrogen exchange data in the region 325–343 indicate that GNF-5 binding alters the protein dynamics in this region. The change can be observed in the raw mass spectra (Fig. S4B), in the deuterium uptake curves (Fig. 2) and in the summary shown in Fig. 3. The change is a decrease in deuterium incorporation, seen clearly for peptide 325–335 in the wild type Abl in the presence of GNF-5 [25] but also for peptides 332–336 and 336–343 in the T315I form. Residues 325–335 correspond to the β-strand that descends down through the center of the β-sheet in the small lobe and emerges on the SH3-side of the ATP binding pocket. This stretch of residues includes the T315I mutation (in Abl 1b numbering, T315I is at position 334, see note above). Many of the amide hydrogens in this region are hydrogen bonded due to participation in the β-sheet of the small lobe, reflected in the small amount of deuterium that is incorporated at early exchange times for peptides in this area. However, this region is dynamic because it can incorporate deuterium over time due to ordinary protein breathing and other motions. The presence of GNF-5 slows these motions and prohibits the incorporation of deuterium in peptides 325–335, 332–336 and 336–343. Although the exact mechanistic details of how distant binding is transmitted through the protein are not revealed by these data, the ability of GNF-5 to inhibit kinase activity on its own in both wild-type and T315I Abl (Fig. 1) must be related to these changes in protein dynamics and point to an allosteric mechanism by which GNF-5 binding is communicated to the ATP site. This does suggest that upon binding of GNF-5 there might be a structural reorganization, possibly communicated via a conformational rearrangement of other parts of Abl, which disrupts the catalytic machinery located in the ATP site.

Choi Y. et al have recently demonstrated that GNF-2 requires Abl SH2 and SH3 domain in order to inhibit kinase activity [44]. Perhaps the GNF class of ligands are able to restore autoinhibition by binding to the myristate pocket and reinducing the kinked conformation of the α-helix to permit docking of the SH2, SH3 domain modules onto the back of the kinase domain. If this were to occur, the autoinhibited conformation would be restored and hydrogen exchange would resemble that of Abl in the downregulated, N-terminally myristoylated state (as examined in [27]). The differences between autoinhibited and active Abl are clear and can be found in the SH2 domain, the linker and parts of the small lobe of the kinase domain [27]. We see no evidence in the current HX MS data for restoration of the autoinhibited form of Abl upon GNF-5 binding, implying that the SH2 and SH3 domains are still displaced form their regulatory positions.

### Conformational changes were additive when both GNF-5 and dasatinib were present

Based on the biological data showing synergy between GNF-5 and ATP-competitive inhibitors (Fig. 1), we examined the effects of binding both dasatinib and GNF-5 to T315I Abl. T315I Abl was incubated with both drugs in a 1∶7∶7 ratio (protein:dasatinib:GNF-5) and deuterium exchange measured. Changes in deuterium uptake were observed in several regions near the ATP binding site as well as by the myristic acid pocket (Fig. 3B). One of the most interesting findings was that a number of peptides in T315I Abl that showed no reduction in deuterium incorporation in the presence of dasatinib alone (Fig. 3A, bottom left) showed nearly the same reduction in deuterium incorporation in dasatinib+GNF-5 (Fig. 3B) as was seen for dasatinib binding to wild-type Abl (Fig. 3A, top left). Reduced deuterium incorporation in the presence of dasatinib+GNF-5 was similar in peptides seen to change in the presence of GNF-5 only (compare dotted curves in Fig. 2), and the only additional peptides seen to be modified over GNF-5 binding alone were 280–288 and 287–298 (Fig. 2). Both 280–288 and 287–298 underwent reduced exchange in wild-type Abl in the presence of dasatinib. Based on these results, we conclude that the effects of dasatinib on Abl conformation and dynamics are restored in the T315I form when GNF-5 is present. For the T315I mutant therefore, as was shown in the biological data, two drugs are better than one and the effects on the conformational dynamics are additive.

### Conclusions

Abl can become resistant to inhibition as a result of ATP site mutations. As shown in our biological assays, alternative inhibitors that target regions of Abl other than the ATP site seem to be effective strategies for overcoming resistance. Our results verified that a double therapy consisting of dasatinib and GNF-5 bound to T315I can synergistically function to inhibit T315I in biochemical and cellular assays, as was also shown for nilotinib and GNF-5 [25]. The question was how? How does binding in distant regions influence kinase activity? We answered the question with HX MS by showing that GNF-5 binding alters the dynamics properties of the ATP site in both wild type and T315I forms of Abl. GNF-5 restores the conformational changes that are seen when dasatinib binds to wild-type Abl. Residues located at positions 325–343 in the ATP site were most influenced by GNF-5 binding and the T315I mutation did not alter the ability of GNF-5 to elicit such changes. These biophysical data should contribute to more rational application of combinations of inhibitors in the challenge to overcome inhibitor resistance.

## Supporting Information

Figure S1 Correct synthesis, purity and post‐translational modifications for each protein were determined with mass spectrometry. The raw m/z data are shown on the left and the transformed, mass only spectra shown on the right. The measured and theoretical molecular weights are indicated. In the transformed mass spectra, P indicates phosphorylation. To obtain these data, approximately 150 pmols of each protein were injected onto a POROS 20 R2 protein trap, desalted with 0.05% trifluroacetic acid (TFA) at 100 μL/min for 2 minutes, and eluted at 50 μL/min with a 4 minute linear 15%–75% (v/v) acetonitrile gradient directly into an LCT‐Premier mass spectrometer (Waters Corp., Milford, MA, USA) equipped with a standard electrospray source. The instrument was calibrated with 500 fmol/µL myoglobin and the mass accuracy was less than 10 ppm. Phosphorylation (+80 or +160 Da) was observed in the intact protein spectra. The location of each phosphorylation was determined by trypsin digestion followed by LC‐MS/MS (data not shown). Each recombinant protein (50 pmol each) was incubated with trypsin (1∶20, trypsin:protein) for 16 hours at 37°C. The resulting peptides were analyzed with a Waters nanoAcquity UPLC system (1.0×100.0 mm ACQUITY C18 BEH column) coupled to a Waters QTof Premier mass spectrometer. Peptide mass spectra were acquired over an m/z range of 100 to 2000. Mass accuracy was ensured by lock‐mass calibration with 100 fmol/µL Glu‐Fibrinogen peptide, and was less than 10 ppm throughout all experiments. MS^E^ was performed on all parent ions, ramping collision energy from 5‐25V. For wild‐type Abl, single phosphorylation corresponded to modification at Tyr412 and double phosphorylation involved Tyr412 and Tyr89. For Abl T315I, single phosphorylation was on Tyr89 and only a small quantity of the molecules contained phosphoryation at both Tyr89 and Tyr412.
(TIF)

Figure S2 GNF‐5 binds to the myristic pocket and inhibits activity in Bcr‐Abl T315I. **A**. Dasatinib antiproliferative EC50 in the presence of 0.4 to 10 μM GNF‐5 on Ba/F3 cells expressing T315I and E505K Bcr‐Abl. **B**. Inhibition of Bcr‐Abl autophosphoryl‐ation was determined by Bcr‐Abl immunoprecipitation, followed by a immunoblot for phospho‐Tyr (Tyr412) [4], phospho‐STAT 5 (Tyr694) and total Bcr‐Abl (antibody K‐12) from cell lystates obtained after treatment of T315I Bcr‐Abl expressing Ba/F3 with 10 μM of dasatinib and increasing concentrations of GNF‐5 (0, 0.5, 5 and 10 μM) for 90 min.
(TIF)

Figure S3 Comparison of deuterium exchange in wild‐type Abl and T315I. The deuterium uptake curves for six representative peptides are shown in the left [solid lines: wild‐type Abl; dotted lines: T315I]. All other deuterium uptake curves for all other regions showed no significance difference between wild‐type and T315I and are therefore not shown. The location of each peptide, according to the labels A‐H, is shown on the crystal structure at the right (PDB 1OPL). Coloring is as in Figure 3: obvious changes (colored hot pink) were defined as a difference between deuterium exchange‐in curves of 1.0 Da or more. Subtle changes (colored light yellow) were 0.4‐1.0 Da. No changes were differences of 0.0‐0.4 Da. Residues corresponding to the hydrophobic spine M309, L320 and F401 are colored blue and rendered as sticks. The 1OPL crystal structure was chosen to display these data because we observed subtle changes in regions outside of the kinase domain, namely in the SH2 domain residues 137‐157. Abl kinase is believed to adopt an extended top‐hat conformation illustrated by this crystal structure (see main text).
(TIF)

Figure S4 Example mass spectra for selected regions, intended to illustrate the quality of data for all experiments. **A**. Residues 287‐298 (peptide m/z =682.3^+2^). **B**. Residues 325‐335 (peptide m/z =683.4^+2^). **C**. Residues 525‐534 (peptide m/z =543.8^+2^). A dotted line is provided at the same m/z in both free or bound data to guide the eye.
(TIF)

Figure S5 Location key for Figure 2 and 3, on PDB 2F4J. Each peptide is colored, according to the scale shown, and the residue numbers indicated [we are numbering according to Abl 1a numbering].
(TIF)

Figure S6 Expanded view of the myristic acid pocket. **A**. Ribbon diagram, **B**. space filling model, in the same orientation as A. This model was created with two crystal structures: PDB 2FO0 and PDB 2F4J were overlaid and aligned. Then, only the αI helix is shown for the 2F4J structure as the rest of the structure was essentially identical to 2FO0. The αI helix for 2F4J is shown in green/blue. In 2FO0, the αI helix is broken into two smaller helices, αI and αI' where an almost 90 degree bend is introduced between αI and αI'. The peptide spanning residues 516‐524 is shown in red (2FO0) or blue (2F4J). Changes in HX are colored as in Figure 3: obvious changes were defined as a difference between deuterium exchange‐in curves of 1.0 Da or more. Subtle changes were 0.4‐1.0 Da. No changes were differences of 0.0‐0.4 Da.
(TIF)
