# Supplementary figures and images for: Allosteric Interactions between the Myristate- and ATP-Site of the Abl Kinase
Source: PLoS One. 2011 Jan 10;6(1):e15929. doi: 10.1371/journal.pone.0015929 (PMC3018526; doi:10.1371/journal.pone.0015929)

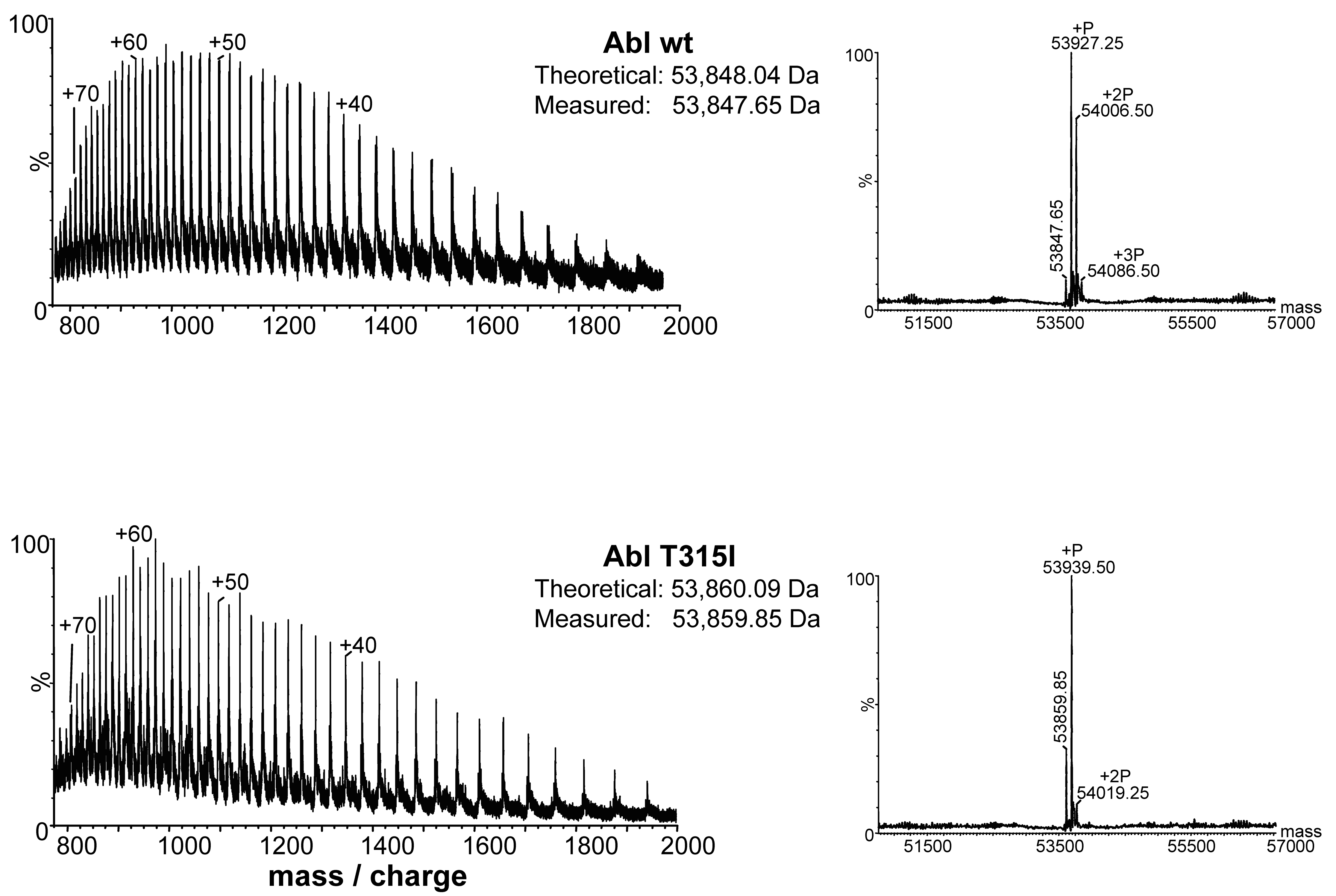

Supplement: Figure S1 — Correct synthesis, purity and post‐translational modifications for each protein were determined with mass spectrometry. The raw m/z data are shown on the left and the transformed, mass only spectra shown on the right. The measured and theoretical molecular weights are indicated. In the transformed mass spectra, P indicates phosphorylation. To obtain these data, approximately 150 pmols of each protein were injected onto a POROS 20 R2 protein trap, desalted with 0.05% trifluroacetic acid (TFA) at 100 μL/min for 2 minutes, and eluted at 50 μL/min with a 4 minute linear 15%–75% (v/v) acetonitrile gradient directly into an LCT‐Premier mass spectrometer (Waters Corp., Milford, MA, USA) equipped with a standard electrospray source. The instrument was calibrated with 500 fmol/µL myoglobin and the mass accuracy was less than 10 ppm. Phosphorylation (+80 or +160 Da) was observed in the intact protein spectra. The location of each phosphorylation was determined by trypsin digestion followed by LC‐MS/MS (data not shown). Each recombinant protein (50 pmol each) was incubated with trypsin (1∶20, trypsin:protein) for 16 hours at 37°C. The resulting peptides were analyzed with a Waters nanoAcquity UPLC system (1.0×100.0 mm ACQUITY C18 BEH column) coupled to a Waters QTof Premier mass spectrometer. Peptide mass spectra were acquired over an m/z range of 100 to 2000. Mass accuracy was ensured by lock‐mass calibration with 100 fmol/µL Glu‐Fibrinogen peptide, and was less than 10 ppm throughout all experiments. MSE was performed on all parent ions, ramping collision energy from 5‐25V. For wild‐type Abl, single phosphorylation corresponded to modification at Tyr412 and double phosphorylation involved Tyr412 and Tyr89. For Abl T315I, single phosphorylation was on Tyr89 and only a small quantity of the molecules contained phosphoryation at both Tyr89 and Tyr412. (TIF) [file pone.0015929.s001.tif]

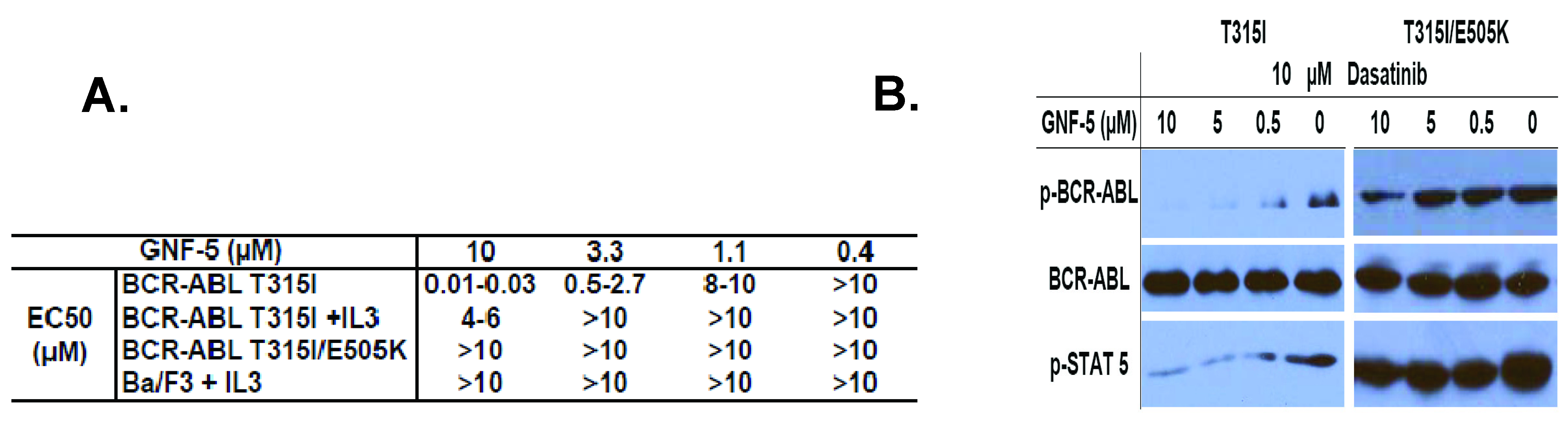

Supplement: Figure S2 — GNF‐5 binds to the myristic pocket and inhibits activity in Bcr‐Abl T315I. A. Dasatinib antiproliferative EC50 in the presence of 0.4 to 10 μM GNF‐5 on Ba/F3 cells expressing T315I and E505K Bcr‐Abl. B. Inhibition of Bcr‐Abl autophosphoryl‐ation was determined by Bcr‐Abl immunoprecipitation, followed by a immunoblot for phospho‐Tyr (Tyr412) [4], phospho‐STAT 5 (Tyr694) and total Bcr‐Abl (antibody K‐12) from cell lystates obtained after treatment of T315I Bcr‐Abl expressing Ba/F3 with 10 μM of dasatinib and increasing concentrations of GNF‐5 (0, 0.5, 5 and 10 μM) for 90 min. (TIF) [file pone.0015929.s002.tif]

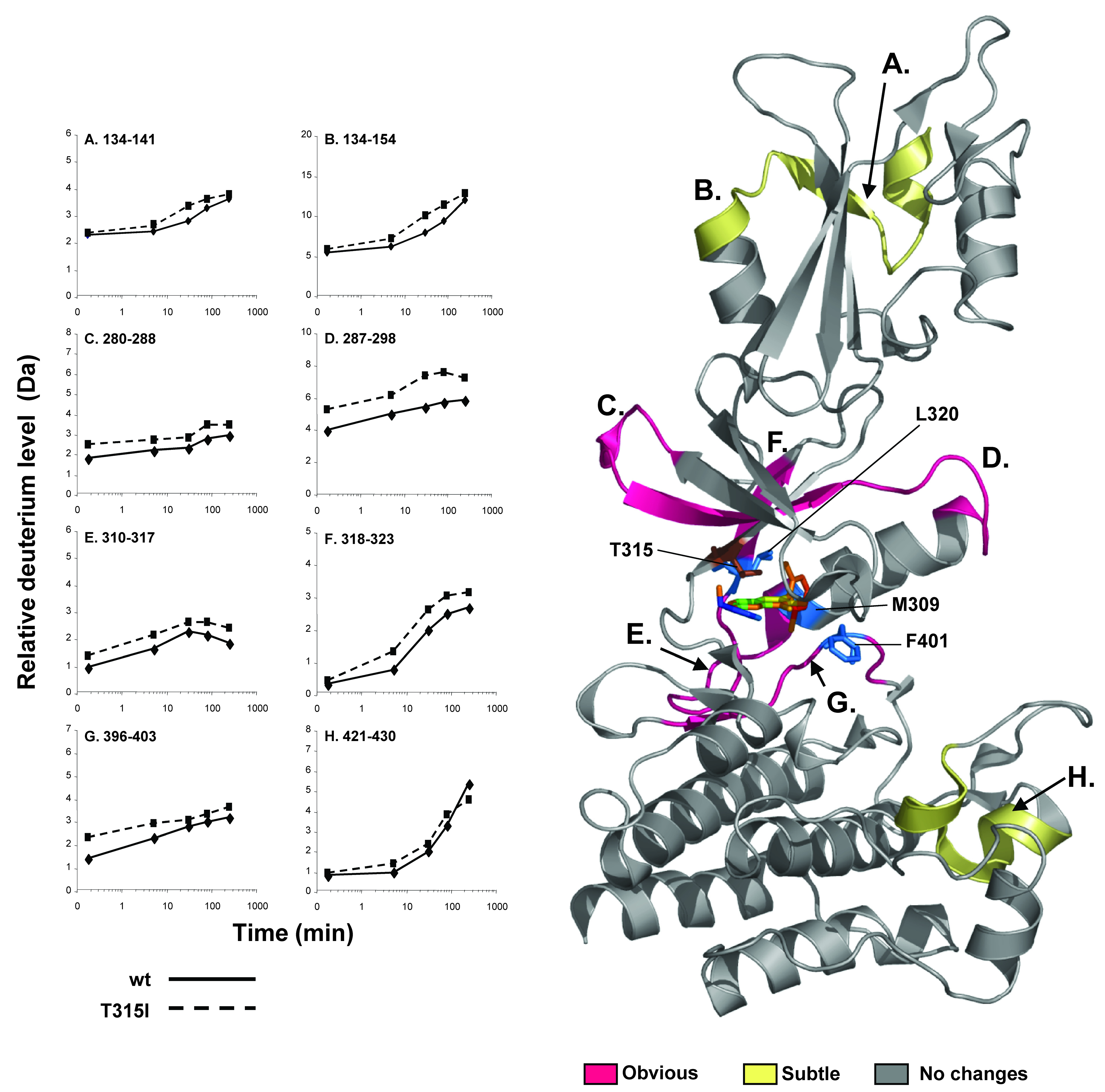

Supplement: Figure S3 — Comparison of deuterium exchange in wild‐type Abl and T315I. The deuterium uptake curves for six representative peptides are shown in the left [solid lines: wild‐type Abl; dotted lines: T315I]. All other deuterium uptake curves for all other regions showed no significance difference between wild‐type and T315I and are therefore not shown. The location of each peptide, according to the labels A‐H, is shown on the crystal structure at the right (PDB 1OPL). Coloring is as in Figure 3: obvious changes (colored hot pink) were defined as a difference between deuterium exchange‐in curves of 1.0 Da or more. Subtle changes (colored light yellow) were 0.4‐1.0 Da. No changes were differences of 0.0‐0.4 Da. Residues corresponding to the hydrophobic spine M309, L320 and F401 are colored blue and rendered as sticks. The 1OPL crystal structure was chosen to display these data because we observed subtle changes in regions outside of the kinase domain, namely in the SH2 domain residues 137‐157. Abl kinase is believed to adopt an extended top‐hat conformation illustrated by this crystal structure (see main text). (TIF) [file pone.0015929.s003.tif]

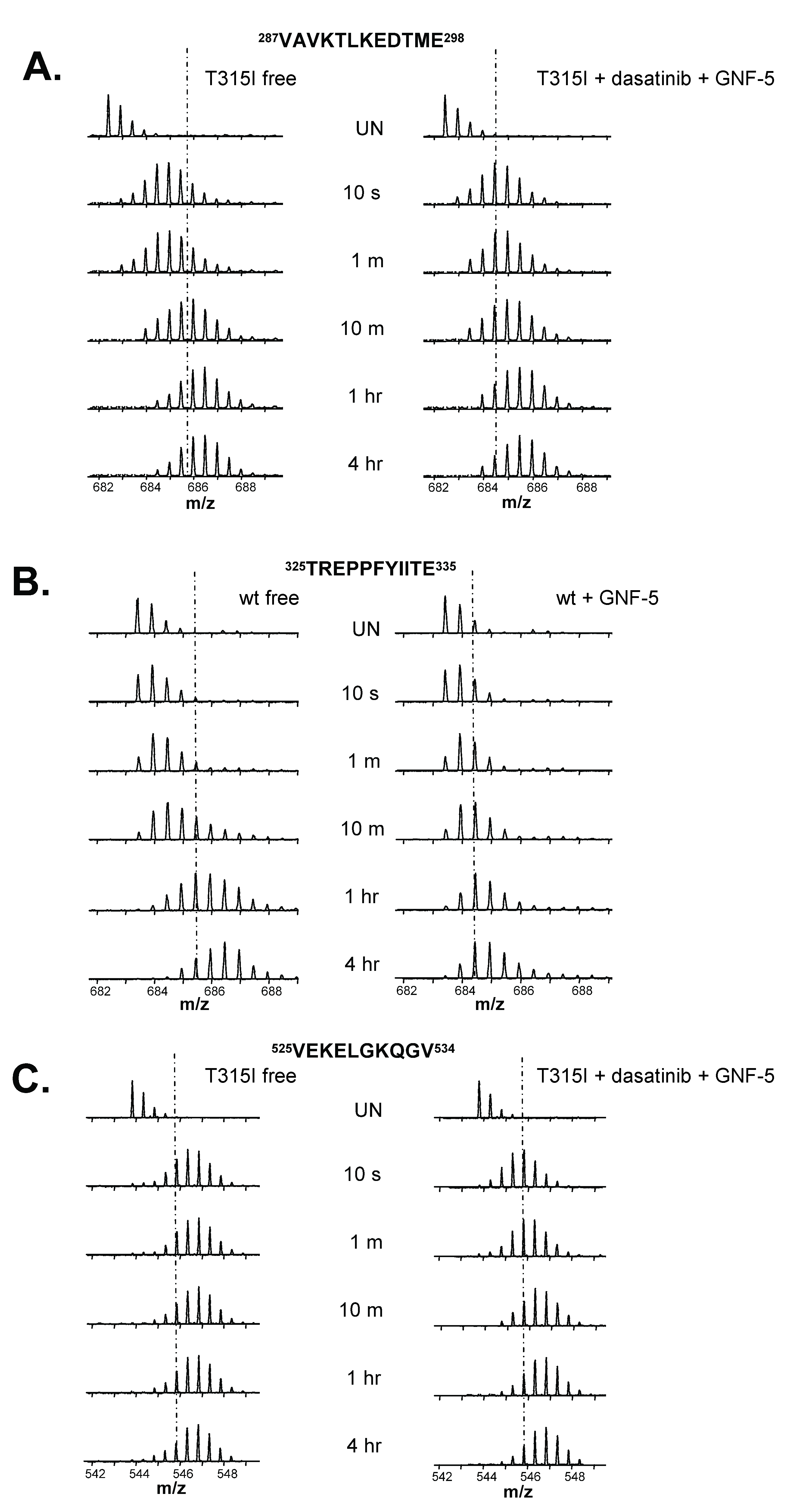

Supplement: Figure S4 — Example mass spectra for selected regions, intended to illustrate the quality of data for all experiments. A. Residues 287‐298 (peptide m/z =682.3+2). B. Residues 325‐335 (peptide m/z =683.4+2). C. Residues 525‐534 (peptide m/z =543.8+2). A dotted line is provided at the same m/z in both free or bound data to guide the eye. (TIF) [file pone.0015929.s004.tif]

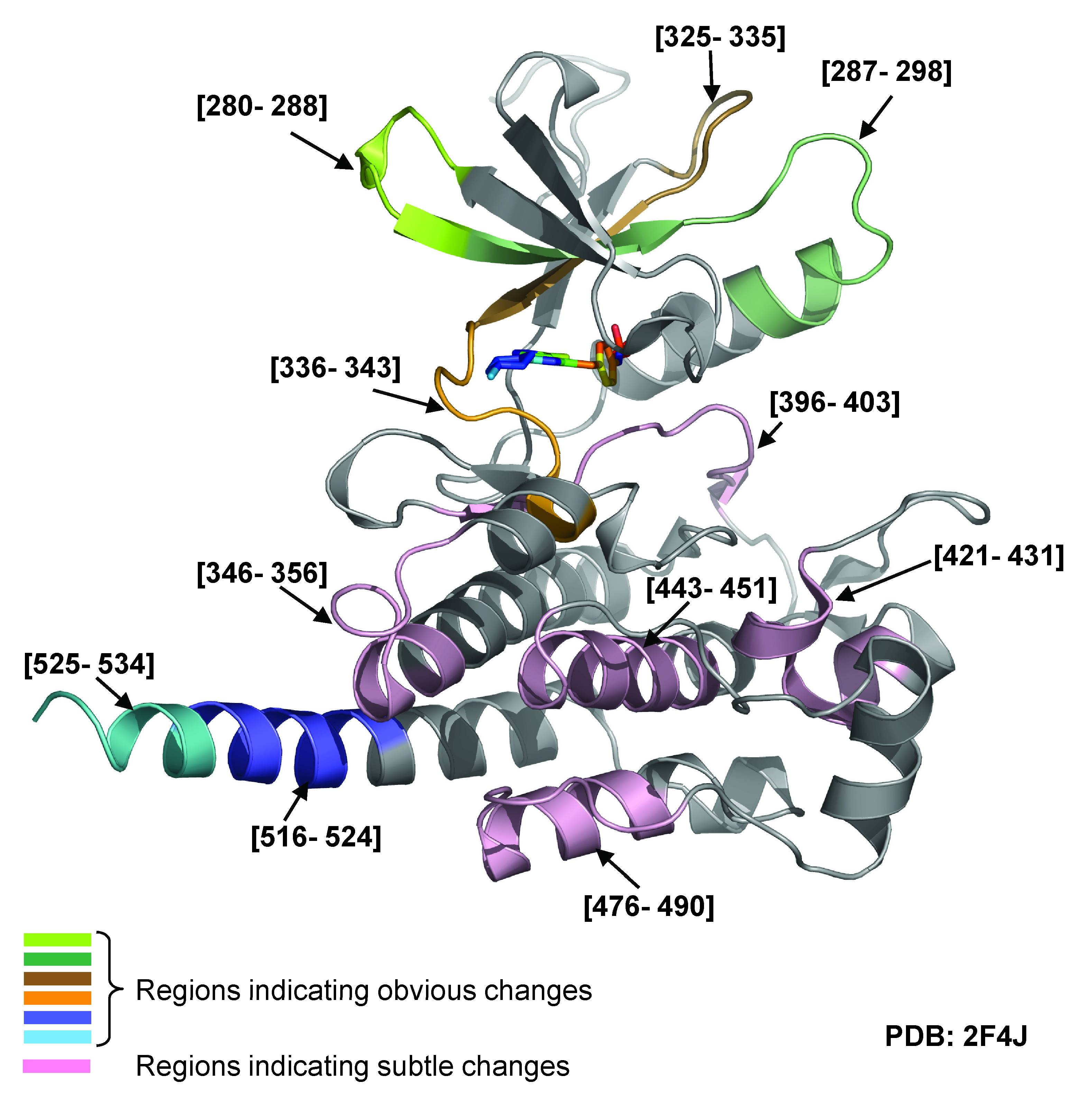

Supplement: Figure S5 — Location key for Figure 2 and 3, on PDB 2F4J. Each peptide is colored, according to the scale shown, and the residue numbers indicated [we are numbering according to Abl 1a numbering]. (TIF) [file pone.0015929.s005.tif]

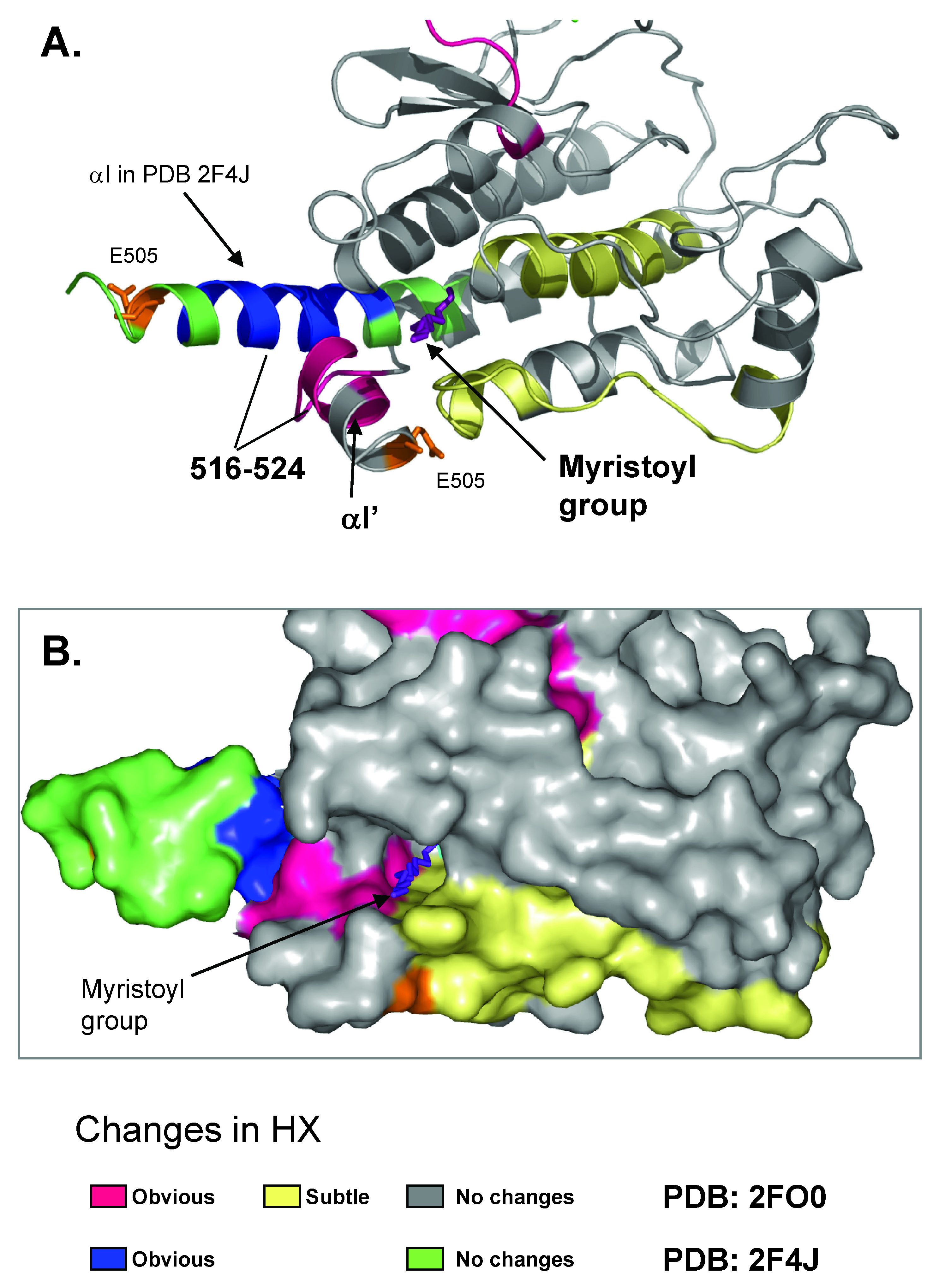

Supplement: Figure S6 — Expanded view of the myristic acid pocket. A. Ribbon diagram, B. space filling model, in the same orientation as A. This model was created with two crystal structures: PDB 2FO0 and PDB 2F4J were overlaid and aligned. Then, only the αI helix is shown for the 2F4J structure as the rest of the structure was essentially identical to 2FO0. The αI helix for 2F4J is shown in green/blue. In 2FO0, the αI helix is broken into two smaller helices, αI and αI' where an almost 90 degree bend is introduced between αI and αI'. The peptide spanning residues 516‐524 is shown in red (2FO0) or blue (2F4J). Changes in HX are colored as in Figure 3: obvious changes were defined as a difference between deuterium exchange‐in curves of 1.0 Da or more. Subtle changes were 0.4‐1.0 Da. No changes were differences of 0.0‐0.4 Da. (TIF) [file pone.0015929.s006.tif]
